# Steroid Versus Non-steroid Intraoperative Multimodal Periarticular Injection in Simultaneous Bilateral Total Knee Arthroplasty Using Real Intelligence CORI: Comparative Observational Study on Postoperative Pain and Early Functional Recovery

**DOI:** 10.7759/cureus.80014

**Published:** 2025-03-04

**Authors:** Avtar K Singh, Arshid H Wani, Babaji Thorat, Vamsi Krishna, Abhijit Das

**Affiliations:** 1 Orthopaedics, Amandeep Hospital, Amritsar, IND; 2 Orthopaedic Surgery, Amandeep Hospital, Amritsar, IND; 3 Orthopedic Surgery, Amandeep Hospital, Amritsar, IND

**Keywords:** bilateral total knee arthroplasty, functional recovery, multimodal, peri articular injection, steroid, visual analog scale (vas) score

## Abstract

Aims and objectives: This study aimed to compare the postoperative pain, early knee range of flexion, and straight leg raise (SLR) outcomes between the right and left knees in patients undergoing simultaneous bilateral primary total knee arthroplasty (TKA). The primary objective of this study was to investigate the comparative effects of a multimodal periarticular cocktail injection injected intraoperatively to both knees, with a specific focus on the impact of adding methylprednisolone to the injection administered to the right knee.

Materials and methods: This prospective observational comparative study enrolled 30 patients (60 knees) who underwent simultaneous bilateral primary TKA. Following strict inclusion and exclusion criteria, all patients were carefully selected for participation. Intraoperatively, a multimodal periarticular cocktail mixture was injected into both knees prior to final implantation. Notably, the right-sided mixture contained methylprednisolone (80 mg). Postoperative follow-up assessments focused on evaluating Visual Analog Scale (VAS) pain scores, degree of range of flexion, and degree of SLR.

Results: The study sample consisted of patients with a mean age of 61.07 years, exhibiting a female-to-male ratio of 16.6:1. Our study demonstrated a significant reduction in VAS scores for the right knee compared to the left knee at all post-operative time points (12, 24, 48, and 72 hours), with p-value < 0.001. Early functional recovery, assessed by knee flexion and SLR, showed improved outcomes in the right knee. At 24, 48, and 72 hours post-operation, the right knee demonstrated a significantly greater range of motion (ROM) compared to the left knee (p < 0.05). The right knee exhibited significantly higher active SLR values than the left knee at 24, 48, and 72 hours post-operation, with statistically significant differences (p < 0.05).

Conclusion: This study demonstrated that the adjunctive use of methylprednisolone in a periarticular intraoperative multimodal cocktail injection significantly improved the postoperative pain and functional outcomes following TKA. Specifically, the addition of methylprednisolone resulted in reduced postoperative pain scores with increased ROM (degrees) and greater active SLR (degrees) compared to the conventional multimodal periarticular cocktail injection.

## Introduction

Approximately 60% of individuals undergoing total knee arthroplasty (TKA) experience intense post-surgical discomfort, whereas 30% endure moderate distress [1]. This affliction can profoundly impact the quality of sleep and impede the resumption of pre-surgical pursuits [2]. Effective modulation of post-surgical discomfort is vital to abbreviate hospitalization, facilitate enhanced convalescence and mobilization, and augment patient gratification. Furthermore, optimal pain control can reduce the risk of post-surgical complications, such as deep venous thrombosis and pneumonitis.

Many pain management modalities are available, e.g., epidural analgesia, regional neural blockade, systemic opioid administration, and continuous intra-articular analgesic infusion. Each modality possesses unique advantages and disadvantages. For instance, epidural analgesia provides effective pain relief but may hinder early mobilization and increase the risk of hypotension, cephalalgia, and spinal infection. Regional neural blockade carries a rare risk of neurovascular trauma, hematoma formation, and infection. Systemic opioid administration can induce nausea, vomiting, respiratory depression, drowsiness, urinary retention, and constipation. Intra-articular analgesic infusion may precipitate joint effusion and increase the risk of infection.

Innovative pain alleviation methodologies have concentrated on modulating nociceptive pathways and inhibiting algoreceptors within the knee joint. This objective is attained through localized periarticular and/or intra-articular injections of analgesic agents [3,4]. This technique boasts several advantages, including fiscal prudence, technical simplicity, and reproducibility. Furthermore, it circumvents motor paralysis and obviates the risk of systemic complications inherent to alternative analgesic modalities. Locally injected methylprednisolone acts by providing anti-inflammatory effects, suppressing leukocyte migration and capillary permeability. This study was performed to compare the effects of additional methylprednisolone in intraoperative periarticular multimodal injection with those of conventional multimodal periarticular injection on postoperative analgesia, early range of motion (ROM), and early active straight-leg raise (SLR) in patients who underwent simultaneous bilateral TKA.

## Materials and methods

This observational study was conducted at Amandeep Hospital, Amritsar, Punjab, India, from March 2024 to October 2024 after ethical approval from the institutional review board of the same hospital (approval letter number AMAN/EC/FEB/24). Written and informed consent was obtained from all the patients. We selected 30 participants (60 knees) of both genders based on specific criteria. Patients eligible for inclusion had grade 3 or 4 osteoarthritis that had not responded to conservative treatment, necessitating bilateral TKA, were aged between 55 and 78 years, and were deemed fit for spinal or epidural anesthesia. Conversely, patients with inflammatory arthritis, a known allergy to local anesthetics or components of the mixture, pre-existing cardiac disorders, diabetes mellitus, compromised immune systems, renal failure, drug addiction, or sagittal and/or coronal knee deformities exceeding 20 degrees were excluded from the present study.

Methods

All patients received spinal anaesthesia and underwent surgery at our specialized hip and knee surgery center, performed by two experienced arthroplasty surgeons. All patients underwent simultaneous TKA of both knees using the real intelligence CORI robotic system by Smith & Nephew, with cemented implants from the same manufacturer. The midvastus approach was used in all the patients in both knees. Intraoperatively, all patients received a periarticular multimodal cocktail injection in both the right and left knee joints. Notably, the right knee received a cocktail mixture containing additional methylprednisolone. The composition of the multimodal cocktail was as follows (Table 1).

**Table 1:** Standard medication protocol for periarticular injection. mg (milligram), ml (millilitre), mcg (microgram), % (percent)

Drug	Dose/strength	Volume
Bupivacaine	5 mg/ml	16 ml
Ketorolac	30 mg/ml	1 ml
Adrenaline	1 mg/ml	1 ml
Fentanyl	50 mcg/ml	2 ml
Normal saline	0.9%	100 ml

Cefuroxime 750 mg was added to the above 120 ml mixture. The total volume of the periarticular cocktail mixture was equally divided for both knees. The right knee injection consisted of the standard cocktail mixture supplemented with 2 ml of methylprednisolone (40 mg/ml), yielding a total steroid dose of 80 mg. Conversely, the left knee received the standard cocktail mixture without any additional methylprednisolone.

Technique

The periarticular cocktail regimen was administered via injections at seven precise anatomical locations surrounding the knee joint immediately prior to implantation, after completion of all bone cuts, knee balancing, and trialing. These zones included Zone 1 (around the medial collateral ligament and at its insertion sites and at the medial meniscus capsular attachment), Zone 2 (within the medial retinaculum), Zone 3 (within the posterior capsule), Zone 4 (around the lateral collateral ligament and its insertion sites and at the lateral meniscus capsular attachment), Zone 5 (within the lateral retinaculum), Zone 6 (the area around the patellar tendon and within the infrapatellar fat pad), and Zone 7 (within the split ends of quadriceps muscle and the area surrounding the quadriceps tendon).

Postoperatively, both knees of each patient were evaluated with the following parameters: Pain intensity was measured using the Visual Analog Scale (VAS; 0-100) at 12, 24, 48, and 72 hours. SLR in degrees was assessed at 24, 48, and 72 hours. The range of knee flexion in degrees was measured using a goniometer at 24, 48, and 72 hours.

The analysis was performed using IBM SPSS Statistics for Windows, version 26.0 (released 2019, IBM Corp., Armonk, NY). Descriptive statistics (mean, median, SD, and range) summarized demographics, ROM, and VAS scores. A paired t-test was applied to assess changes in ROM, VAS, and SLR between the right and left knees over time, and bar diagrams were used to present the data. 

## Results

The study included 30 participants, predominantly female (25 participants, 83.33%) compared to male (five participants, 16.67%). The participants ranged from 55 to 78 years, with a mean age of 61.07 (SD = 8.09) and a median age of 60.50.

**Figure 1:**
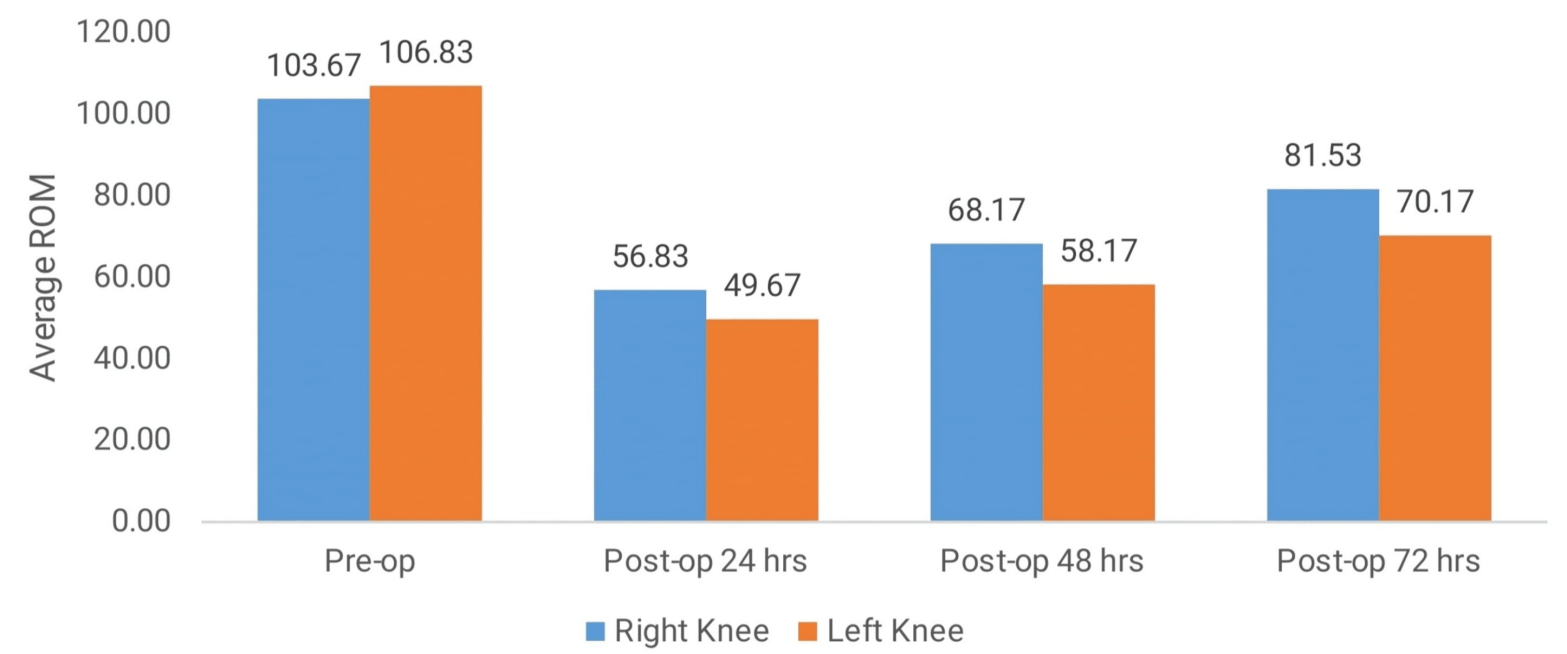
Comparison of ROM (degrees) in the right and left knees at different intervals. ROM (range of motion)

The table below shows the comparison of ROM in the right and left knees at different postoperative intervals (Table 2). Preoperatively, the ROM (degrees) was slightly higher in the left knee (106.83 ± 12.49) compared to the right knee (103.67 ± 12.03), but the difference was not statistically significant (p = 0.05, NS). At 24 hours post-operation, the right knee showed a higher ROM (56.83 ± 15.17) compared to the left knee (49.67 ± 15.81), with p-value = 0.021. This difference widened at 48 hours post-operation, where the ROM of the right knee (68.17 ± 13.23) was significantly greater than the left knee (58.17 ± 16.05), with a significant p-value of 0.002. By 72 hours post-operation, the trend continued, with the right knee (81.53 ± 12.86) showing significantly better ROM than the left knee (70.17 ± 16.32), reflected in a p-value of 0.004. These findings suggest that the right knee consistently achieved better ROM recovery compared to the left knee during the postoperative period (Figure 1).

**Table 2:** Comparision of ROM (degrees) in the right and left knees at different intervals. ROM (range of motion), N (number of patients), SD (standard deviation), SEM (standard error of the mean), t (Student's t-test), df (degrees of freedom), p-value (probability value)

		Mean	N	SD	SEM	t	df	p-value
Pre-op	Right knee	103.67	30	12.030	2.196	-2.129	29	0.05, NS
	Left knee	106.83	30	12.490	2.280			
Post-op 24 hours	Right knee	56.83	30	15.170	2.770	2.435	29	0.021
	Left knee	49.67	30	15.808	2.886			
Post-op 48 hours	Right knee	68.17	30	13.228	2.415	3.501	29	0.002
	Left knee	58.17	30	16.054	2.931			
Post-op 72 hours	Right knee	81.53	30	12.862	2.348	3.127	29	0.004
	Left knee	70.17	30	16.320	2.980			

**Figure 2:**
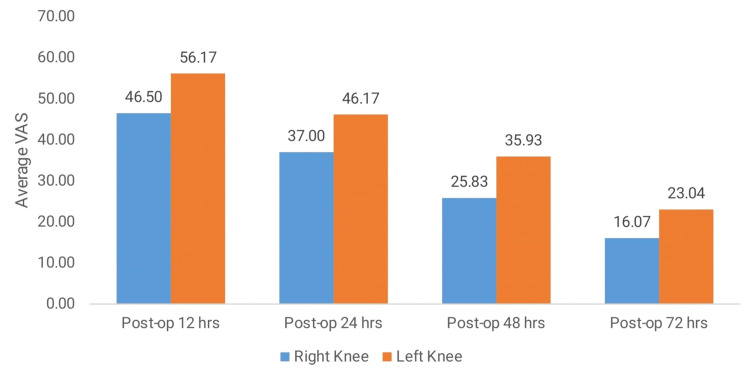
Comparison of VAS score in the right and left knees at different intervals. VAS (Visual Analogue Scale)

The comparison of the VAS scores for pain between the right and left knees at various postoperative intervals indicated that the right knee consistently experienced significantly lower pain levels than the left knee. At 12 hours post-operation, the VAS score for the right knee was 46.50 ± 11.15 compared to 56.17 ± 10.48 for the left knee, with a p-value of <0.001. This pattern continued at 24 hours post-operation, with the right knee exhibiting a significantly lower pain score (37.00 ± 9.06) compared to the left knee (46.17 ± 11.42, p < 0.001). By 48 hours post-operation, pain levels further decreased in the right knee (25.83 ± 9.29) relative to the left knee (35.93 ± 11.23), maintaining a significant p-value of <0.001. By 72 hours post-operation, the VAS scores were the lowest, with the right knee scoring 16.07 ± 9.06 and the left knee 23.04 ± 10.03, still showing a significant difference (p < 0.001). These results suggest that the right knee consistently reported less pain than the left knee during the entire postoperative period (Table 3, Figure 2).

**Table 3:** Comparision of VAS scores in right and left knee at different intervals. VAS (Visual Analogue Scale), N (number of patients), SD (standard deviation), SEM (standard error of the mean), t (Student's t-test), df (degrees of freedom), p-value (probability value)

		Mean	N	SD	SEM	t	df	p-value
Post-op 12 hours	Right knee	46.50	30	11.153	2.036	-4.229	29	<0.001
	Left knee	56.17	30	10.478	1.913			
Post-op 24 hours	Right knee	37.00	30	9.059	1.654	-4.504	29	<0.001
	Left knee	46.17	30	11.423	2.086			
Post-op 48 hours	Right knee	25.83	30	9.293	1.697	-4.355	29	<0.001
	Left knee	35.93	30	11.231	2.050			
Post-op 72 hours	Right knee	16.07	30	9.063	1.713	-3.978	29	<0.001
	Left knee	23.04	30	10.031	1.896			

**Figure 3:**
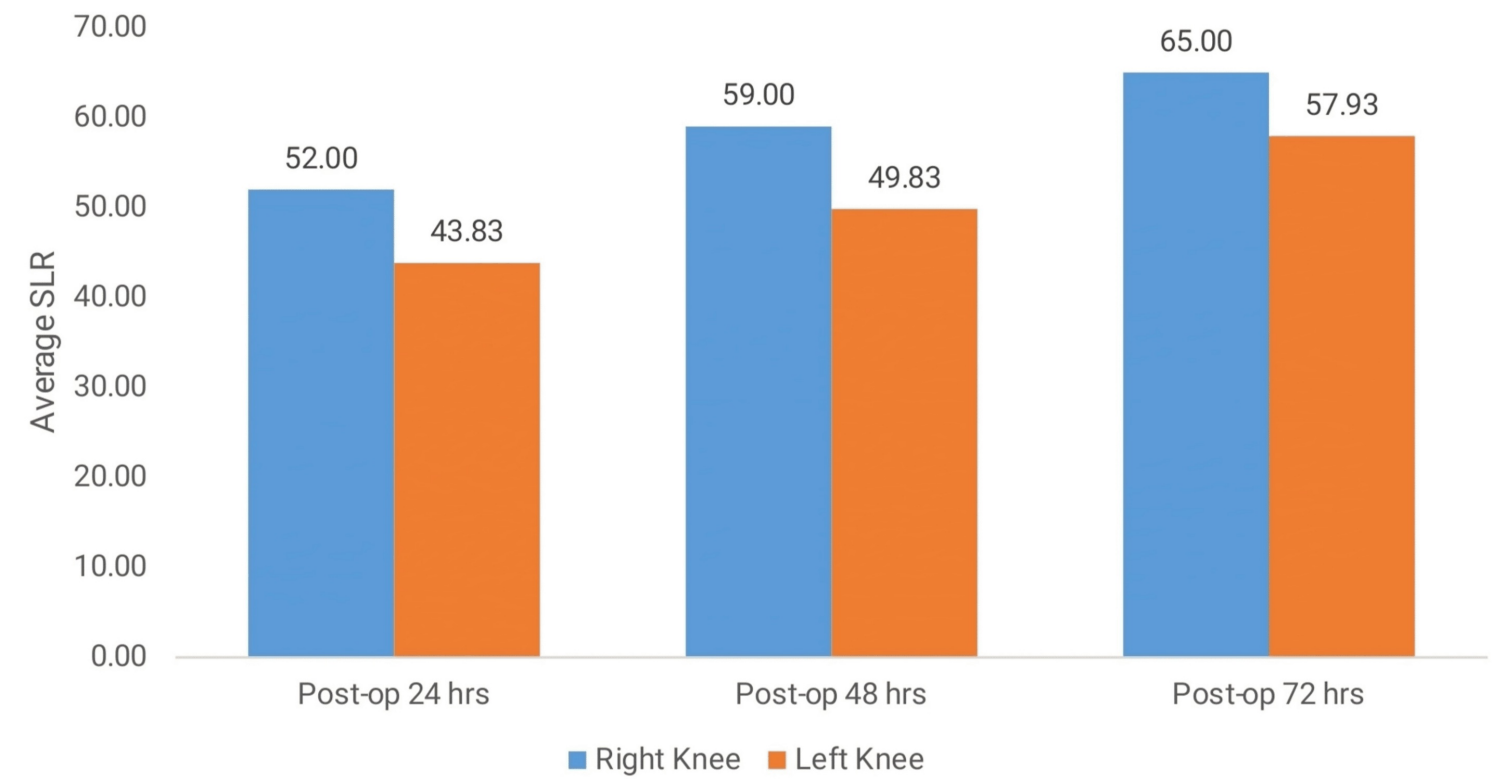
Comparison of SLR in the right and left knees at different intervals. SLR (straight leg raise)

The comparison of SLR (degrees) between the right and left knees at different postoperative intervals showed significantly better performance in the right knee across all time points. At 24 hours post-operation, the mean SLR for the right knee (52.00 ± 13.93) was significantly higher than that of the left knee (43.83 ± 15.07), with a p-value of 0.002. This improvement continued at 48 hours post-operation, with the right knee achieving a mean SLR of 59.00 ± 11.99 compared to 49.83 ± 13.49 for the left knee, showing a highly significant p-value of <0.001. By 72 hours post-operation, the right knee showed further improvement in SLR (65.00 ± 10.52) compared to the left knee (57.93 ± 11.54), with a p-value of 0.001. These findings indicated that the right knee consistently exhibited superior recovery in SLR compared to the left knee during the postoperative periods (Table 4, Figure 3).

**Table 4:** Comparison of SLR (degrees) in the right and left knees at different intervals. SLR (straight leg raise), N (number of patients), SD (standard deviation), SEM (standard error of the mean), t (Student's t-test), df (degrees of freedom), p-value (probability value)

		Mean	N	SD	SEM	t	df	p-value
Post-op 24 hours	Right knee	52.00	30	13.933	2.544	3.365	29	0.002
	Left knee	43.83	30	15.068	2.751			
Post-op 48 hours	Right knee	59.00	30	11.991	2.189	4.051	29	<0.001
	Left knee	49.83	30	13.486	2.462			
Post-op 72 hours	Right knee	65.00	30	10.522	1.954	3.858	29	0.001
	Left knee	57.93	30	11.535	2.142			

## Discussion

Pain management following TKA is a paramount concern. Current pain management protocols primarily focus on mitigating pain sensitization by ensuring uninterrupted analgesic coverage throughout the patient's hospitalization period. Although epidural analgesia is the most widely employed modality for pain control, it is often complicated by adverse events such as headache, hypotension, respiratory depression, and cardiac decompensation [5,6]. Regional nerve blocks, including femoral nerve and adductor canal blocks, carry a 1-2.5% risk of nerve injury, muscle weakness, and localized infection. Furthermore, these catheters are susceptible to bacterial colonization within 48 hours, occurring in up to 57% of cases [7,8].

The injection of a combination of therapeutic agents into the periarticular region is a vital component of the multimodal strategy, playing a key role in optimizing postoperative pain control [9,10]. Busch et al. conducted a landmark study on periarticular infiltration analgesia for TKA. Their findings revealed that patients treated with a combination of ketorolac, ropivacaine, morphine, and epinephrine required substantially less supplemental pain medication and experienced a prolonged opioid-free postoperative period [11]. Thorsell et al. observed that patients who underwent local infiltration analgesia experienced faster postoperative pain relief and mobilization compared to those who received epidural anesthesia [12]. Nair et al. conducted a study in which patients undergoing TKA received either a cocktail injection comprising normal saline, bupivacaine, ketorolac, and adrenaline or a control injection consisting of an equivalent volume of normal saline. The results showed that the cocktail injection group experienced significantly reduced post-operative pain and improved range of motion compared to the control group [13]. The addition of epinephrine to the local anesthetic solution helped minimize systemic toxicity by restricting the spread of the anesthetic [14].

The composition of our periarticular analgesic cocktail in patients who underwent simultaneous bilateral TKA for bilateral knee analgesia included bupivacaine, ketorolac, epinephrine, fentanyl, and normal saline. Methylprednisolone was added to the cocktail for the right knee, whereas the left knee received the same formulation without methylprednisolone, which served as a control, highlighting the comparative benefits of methylprednisolone. Bupivacaine, a well-established long-acting local anesthetic, was combined with epinephrine to minimize systemic toxicity and prolong its duration of action. Epinephrine induces vasoconstriction and also helps minimize intra-articular bleeding. Ketorolac is a non-selective cyclooxygenase (COX) inhibitor that blocks the conversion of arachidonic acid into pro-inflammatory mediators, including thromboxane, prostacyclin, and prostaglandins. By inhibiting this pathway, ketorolac reduces the sensitization of afferent nerves, thereby decreasing pain transmission. Fentanyl is an opioid medication that primarily acts through μ opioid receptors. Methylprednisolone acts by providing anti-inflammatory effects, suppressing leukocyte migration and capillary permeability. The suppression of inflammatory reaction by periarticular steroid injection is explained by the cascade inhibiting the cytoactivity of immunocytes by blocking the synthesis of phospholipase A2 and thereby reducing the production of inflammatory mediators and cytokines [15].

Our study, conducted in patients undergoing simultaneous bilateral TKA, showed that the addition of methylprednisolone to the multimodal periarticular analgesic cocktail, injected intraoperatively into the right knee, resulted in significantly reduced postoperative pain intensity in the same knee, as measured by the VAS, compared to the left knee. Furthermore, early postoperative functional recovery was enhanced, with improved SLR and ROM, compared to the left knee. A review of the literature reveals a mixed evidence base regarding the efficacy of corticosteroids (methylprednisolone) in periarticular injections. Research by Ng et al. [16] and Ikeuchi et al. [17] highlighted the anti-inflammatory properties of steroids, both locally and systemically, as indicated by decreased levels of interleukin-6 and C-reactive protein. Concerns about postoperative infection and wound complications have led many surgeons to exercise caution and avoid using periarticular steroids [18,19]. Three randomized controlled trials (RCTs) support the use of methylprednisolone in periarticular injection in TKA, demonstrating its effectiveness in reducing pain and inflammation [17,20,21]. Conversely, two RCTs failed to demonstrate a significant benefit [22,23]. A meta-analysis conducted by Xinyu Zhao et al. revealed that the addition of steroids to multimodal cocktail periarticular injections (MCPI) does not increase the risk of postoperative infections or wound complications. Furthermore, none of the included studies reported any cases of tendon rupture [24]. We did not observe any cases of postoperative infection, wound complications, or tendon rupture.

This study had two main limitations. First, it was conducted in a single center. Second, the sample size was small, making it difficult to draw definitive conclusions about the ratio of complications, including surgical site soakage and wound complications. This observational study had only 30 patients. Furthermore, well-designed and adequately powered studies need to be done to investigate our findings. To analyze the impact of corticosteroids on surgical site infections, a larger sample size is needed. The strength of our study lies in its novelty, as it is the first study to evaluate the effects of additional methylprednisolone in periarticular cocktail injections administered intraoperatively around the knee during simultaneous bilateral TKA. Notably, the same patient acted as a control, which helped to minimize some of the confounding factors.

## Conclusions

Our study, conducted in patients who underwent simultaneous bilateral TKA, demonstrated that the addition of methylprednisolone to a periarticular multimodal cocktail mixture, injected intraoperatively around the right knee, significantly improved postoperative pain control and resulted in early functional recovery, compared to the left knee, which received a standard cocktail mixture. These findings suggest that adjunctive steroid use in multimodal periarticular injections is a safe and effective strategy for mitigating postoperative pain and promoting early recovery. The results provide valuable insights into the therapeutic potential of steroids in periarticular injections, informing the development of optimized analgesia protocols for TKA patients.
